# Juvenile granulosa cell tumor of the testis: prenatal diagnosis and prescrotal approach

**DOI:** 10.1186/1824-7288-38-67

**Published:** 2012-12-06

**Authors:** Anna Lavinia Bulotta, Francesco Molinaro, Rossella Angotti, Francesco Ferrara, Giovanni Di Maggio, Edoardo Bindi, Mario Messina

**Affiliations:** 1 Division of Pediatric Surgery, Department of Pediatrics, Obstetrics and Reproductive Medicine, University of Siena, Siena, Italy

**Keywords:** Gonadal sex cord stromal tumor, Orchiectomy, Alpha fetoprotein, Ultrasound, Human chorionic gonadotropin

## Abstract

Neonatal testicular tumors are rare and should be considered in the differential diagnosis of newborn scrotal masses. Juvenile granulosa cell tumor (JGCT) accounts for about 5% of all prepubertal testis tumors. As a benign neoplasm, radical orchiectomy is sufficient for treatment. We report a case of a newborn with a prenatal diagnosis of scrotal mass. After surgery, the histological diagnosis was juvenile granulosa cell tumor. To date the patient is healthy.

## Introduction

Prepubertal testicular tumors are rare and they account for 1% of all pediatric solid tumors. Granulosa cell tumor is a benign neoplasm, and it can be distinguished by adult or juvenile type, with the juvenile type accounting for 1% to 5% of all prepubertal testis tumors. Although rare, it is the most common stromal cord neoplasm of the testis in infancy [1]. JGCT typically presents a painless scrotal mass. Ultrasound evaluation typically shows a circumscribed tumor with multicystic appearance [2]. Only one case of prenatal diagnosis has been reported [3]. Although inguinal orchiectomy has been the standard surgical therapy, a testicular sparing surgery has been recently proposed but it is still not general practice among pediatric surgeons. We reported a case of JGCT with prenatal diagnosis treated by an orchiectomy performed with a prescrotal approach.

### Case report

**Figure 1:**
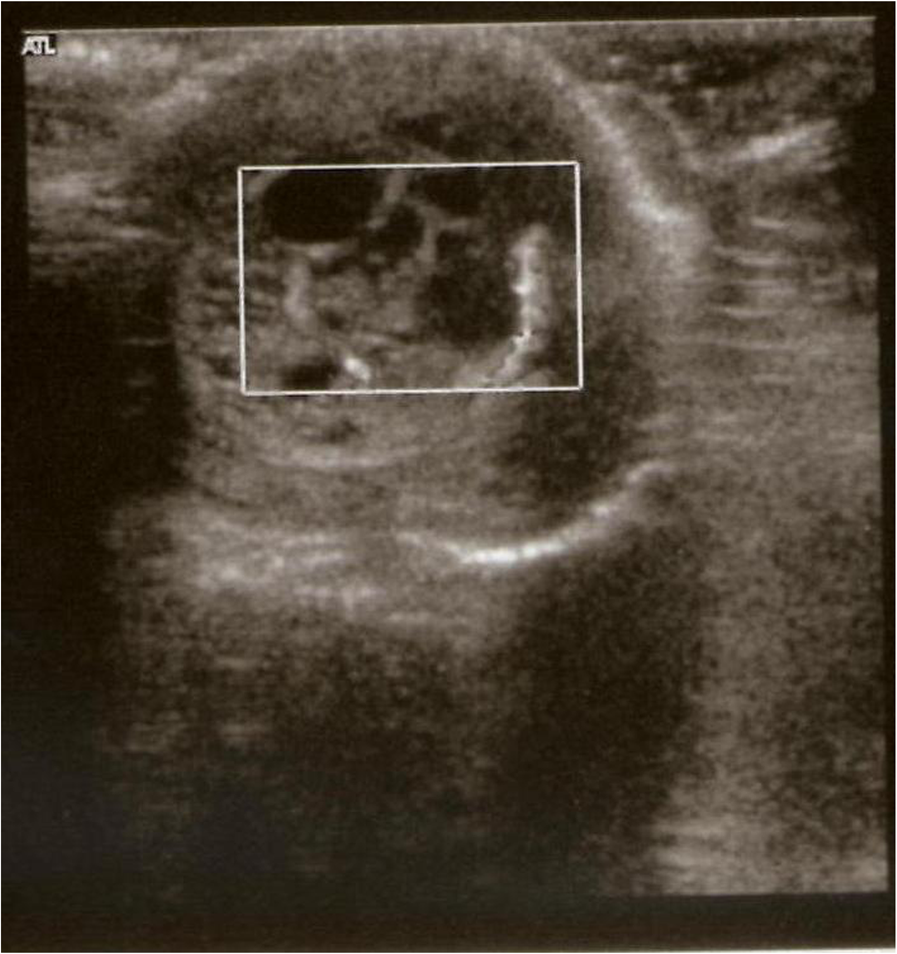
Ultrasound image obtained at birth revealing enlarged left testicle (1.57 × 1.33 cm) and a well encapsulated, complex cystic mass.

**Figure 2:**
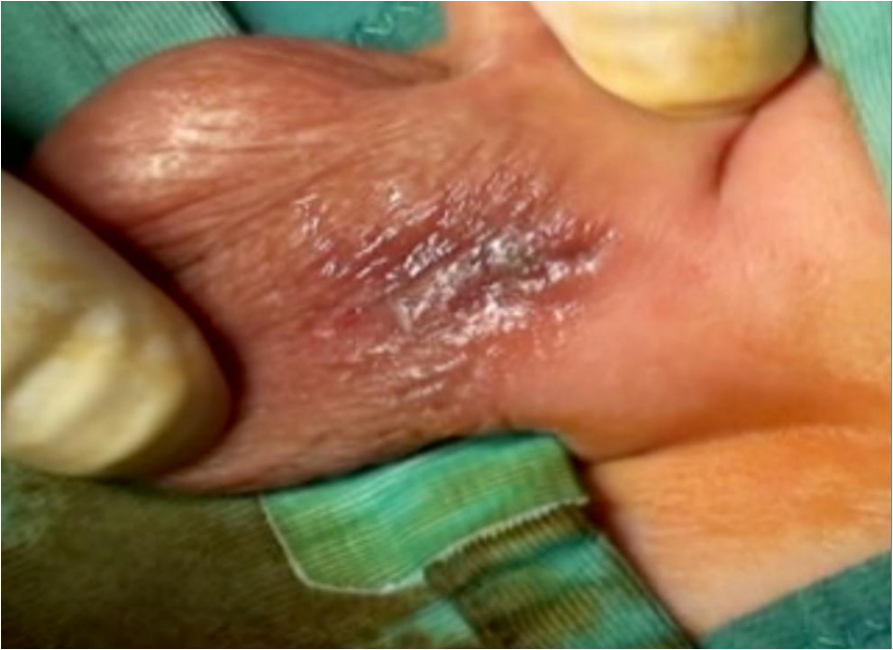
Prescrotal skin incision.

**Figure 3:**
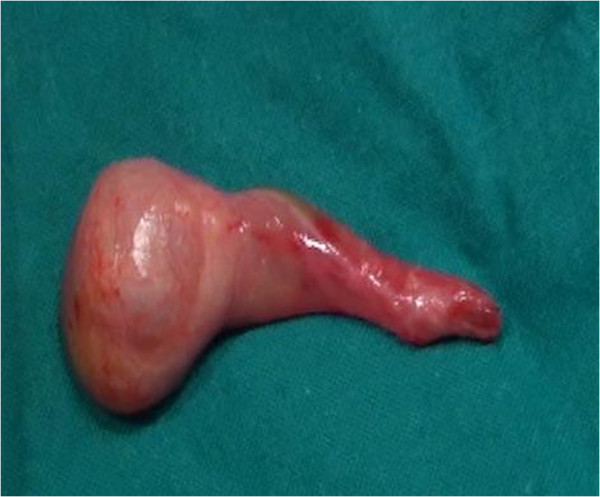
Macroscopic features of the testicle after surgery.

A full term baby was born by natural delivery with a normal APGAR score and a birth weight of 3200 grams. Fetal ultrasound at 36^th^ week of gestation showed a multicystic testicular mass on the left side. Postnatal ultrasound revealed increased size of the testicle (1.57 × 1.33 cm) due to a well encapsulated, complex cystic mass and a Doppler scan showed increased blood flow. The controlateral gonad was normal and showed homogeneous echotexture. After the birth, the baby was admitted to our department for the management of a "left scrotal mass". Family history included two cases of Down's syndrome and no other urogenital problems. The baby was in good health and without clinical evidence of dysmorphic features or ambiguous genitalia. The right testicle had descended, hypoplastic and the penis was normal. The left testicle was in situ and painless, with increased size and consistency. Bilateral inguinal hernia and palpable lymphadenopathy were absent. We performed ultrasonography of the abdomen and testis, as well as karyotype, blood chemistry and hormone profile. The abdominal ultrasound, blood chemistry and karyotype were normal. Testicular scans confirmed the presence of a cystic mass in the left gonad (Figure 1). Abnormal serum tumor markers were: alphafetoprotein (AFP) 6679 IU/ml, human chorionic gonadotropin 8 mIU/ml, testosterone 1.75 ng/ml and inhibin B 346 pg/ml. After long discussion with the parents, it was decided to perform left radical orchiectomy by sub-inguinal (prescrotal) skin incision (Figure 2). Macroscopic features of the testis suggested a neoplastic mass (Figure 3). The gonad had increased size with a pearly white surface and apparently complete capsule. No torsion of the tumor was found. Histological examination showed a juvenile granulosa cell tumor with immunohistochemical stain positive for inhibin and calretinin and negative for AFP. When incised, the gonad was spongy and yellow with many microcysts and no normal testicular parenchyma. There were no intra or postoperative complications. The baby was discharged three days later. Testicular and abdominal ultrasonography a month after surgery showed an empty left scrotum and an absence of pathological masses in the abdomen. Today the patient is healthy without any problems. It is planned to check the patient every year for at least 5 years. The parents were informed of the prognosis and the ongoing clinical examinations and tests required.

## Discussion

The granulosa cell tumor (GCT) is a distinctive form of gonadal sex cord stromal tumor and accounts for 1-5% of all prepubertal testis tumors. It is a benign neoplasm, hormonally inactive and its prognosis is very good.

Although usually diagnosed in the first year of life with a typical clinical presentation characterized by a painless testicular mass that may involve an undescended or scrotal testis, some authors reported a prenatal diagnose of testicular mass. Peterson and Skoog reported a case of a cystic structure seen with an ultrasound evaluation at 35 week’s gestation [3]. In our case fetal ultrasound at 36^th^ week of gestation showed a multicystic alteration of the left testicle. As confirmed by our case, prenatal diagnosis is difficult but possible. Evidence of a general testicular alteration in the unborn baby enabled early diagnosis at birth by clinical examination. Sometimes the tumor presents concomitantly with neonatal torsion [2,4].

Our patient was a neonate and the testis involved was in situ, without torsion. As the tumor may be associated with anomalous karyotype or ambiguous genitalia [5,6] we performed a genetic test, which revealed a normal karyotype, and the clinical features of the genitalia were normal.

Postnatal management consisted of abdominal and testicular ultrasound, karyotyping, blood chemistry and hormonal evaluation. Abdominal ultrasound was negative, whereas testicular scans showed a left testicle of increased size and echotexture.

In our practice we use to determine serum AFP and B-HCG in all infants with a testicular tumors. In our case serum levels of AFP and B-HCG were elevated at preoperative evaluation, according to the reference range for normal AFP established by WU et al. [7], as many Authors recognize the normal serum AFP range for neonates varies according to gestational age and body weight and it is physiologically elevated in neonatal age [8]. Serum AFP in the normal range for age reliably excludes a yolk sac tumor and for this reasons some authors reported that this fact indicates that a testis sparing procedure is feasible [9]. As stated by Grady et al., normal AFP level should not be expected in infants with benign teratoma [10].

In our case we decided to perform a total orchiectomy because of the presence of elevated values of serum AFP. We performed a subinguinal approach with the possibility to realize a safe orchiectomy with an high clamping of spermatic vessels. This approach is less invasive as an inguinal approach and it allows to perform a safe orchiectomy. This incision enables a good visualization of the testicle and spermatic cord, allowing complete excision of the intact mass and thorough assessment of the margins for partial resection if possible.

Chemotherapy and radiotherapy are not contemplated for this tumor because it is non invasive and metastases have never been described.

Follow-up is a controversial topic; some authors suggest that it should be performed for at least 5 years after surgery, as for most other tumors. Others argue that because orchiectomy is the only treatment, no further management is required after surgical removal of the testicle [5]. However, we decided to evaluate the patient one and three months after the surgery, with blood tests and testicular ultrasound. We suggested annual ultrasonography and clinical evaluation for at least 5 years after surgery.

## Conclusion

We agree with other reports that although neonatal testicular tumors are rare, they should be considered in the differential diagnosis of scrotal masses in newborns. Testicular masses should be considered pathological until proven otherwise and a prenatal diagnosis of a scrotal mass is possible. We therefore recommend thorough inspection of the genitalia at birth, followed if necessary by hormonal assessment and testicular ultrasound. In patients affected by a testicular neoplastic disease, we argue that the only possible approach is inguinal. A subinguinal approach enables the testicle and spermatic cord to be exposed, allowing complete excision of the intact mass and thorough assessment of the margins for partial resection if possible. Follow-up of the present case is still too short to exclude recurrence, although no cases of recurrence have been reported in literature. In conclusion, the main aim of our follow-up of this patient will be to avoid damage to the surviving testicle, protect fertility and monitor for eventual recurrence.

Written informed consent was obtained from the patients parents for publication of this report and any accompanying images.

## Competing interest

The authors declare that they have no competing interests.

## Authors’ contributions

All authors read and approved the final manuscript.
